# Social Media Use, Physical Activity, and Internalizing Symptoms in Adolescence: Cross-sectional Analysis

**DOI:** 10.2196/26134

**Published:** 2021-09-15

**Authors:** Lauren A Rutter, Holly M Thompson, Jacqueline Howard, Tennisha N Riley, Robinson De Jesús-Romero, Lorenzo Lorenzo-Luaces

**Affiliations:** 1 Department of Psychological and Brain Sciences Indiana University Bloomington Bloomington, IN United States; 2 Department of Counseling and Educational Psychology Indiana University Bloomington Bloomington, IN United States

**Keywords:** social media, depression, anxiety, physical activity, adolescence, mobile phone

## Abstract

**Background:**

Most American adolescents have access to smartphones, and recent estimates suggest that they spend considerable time on social media compared with other physical and leisure activities. A large body of literature has established that social media use is related to poor mental health, but the complicated relationship between social media and symptoms of depression and anxiety in adolescents is yet to be fully understood.

**Objective:**

We aim to investigate the relationship between social media use and depression and anxiety symptoms in adolescents by exploring physical activity as a mediator.

**Methods:**

A Qualtrics survey manager recruited adult panel participants between February and March 2019, who indicated that they had adolescent children who spoke English. A total of 4592 adolescent-parent dyads completed the survey that took approximately 39 minutes. The survey entailed completing web-based questionnaires assessing various aspects of social media use, psychological symptoms, and psychosocial factors. The average age of the adolescent participants was 14.62 (SD 1.68; range 12-17) years, and the majority of the adolescent sample was male (2392/4592, 52.09%).

**Results:**

Total social media use was associated with more depressive symptoms (multiple *R^2^*=0.12; *F*_3,4480_=207.1; *P*<.001), anxiety (multiple *R^2^*=0.09; *F*_3,4477_=145.6; *P*<.001), and loneliness (multiple *R^2^*=0.06; *F*_3,4512_=98.06; *P*<.001), controlling for age and gender. Physical activity was associated with decreased depression and anxiety symptoms after controlling for other extracurricular activities and social media use (multiple *R^2^*=0.24; *F*_5,4290_=266.0; *P*<.001). There were significant differences in symptoms based on gender: female adolescents reported higher rates of social media use and males reported higher rates of depression. Nonbinary and transgender adolescents had higher rates of depression, anxiety, and loneliness than the female and male adolescents in the sample.

**Conclusions:**

In a nationally representative sample of adolescents, more social media use was associated with more severe symptoms of depression, anxiety, and loneliness. Increased physical activity was associated with decreased depression and anxiety symptoms. Physical activity partially mediated the relationship between social media use and depression and anxiety. As this was a cross-sectional study, we cannot conclude that social media use causes internalizing symptoms or that physical activity leads to decreased internalizing symptoms—there may be additional confounding variables producing the relationships we observed. Physical activity may protect against the potentially harmful effect of social media on some adolescents. The effect sizes were small to medium, and the results should be interpreted with caution. Other limitations of this study include our reliance on self-reporting. Future work should examine social media use beyond how much time adolescents spend using social media and instead focus on the nature of social media activity.

## Introduction

### Background

Social media refers to web-based networks that enable users to interact with each other visually and verbally [1] via a public or semipublic profile within a bounded system [2]. Social media is ubiquitous, and its use continues to rise, especially among adolescents and young adults. Over a span of 6 years, the number of adolescents with smartphones grew from 4 in 10 adolescents to 9 in 10 adolescents [3]. In the same span of time, the percentage of adolescents using social media more than once a day doubled from 34% to 70%. A 2018 study conducted by the Pew Research Center found that 45% of teenagers reported being online on a “near-constant” basis [3,4]. Thus, it is important to understand the associations between social media use and adolescents’ developmental and mental health outcomes, while also considering alternative activities (physical activities and extracurricular activities) intended to promote adolescent health. Moreover, although there is substantial evidence that physical activity is a protective factor against the development of internalizing disorders (depression and anxiety disorders) in adults with clinical and nonclinical levels of depression and anxiety [5], less is known about the connection between physical activity, other extracurricular activities, social media use, and internalizing symptoms (depression and anxiety) during adolescence.

### Physical Activity (Exercise) and Internalizing Symptoms in Adolescence

In a study conducted by Bartels et al [6] to examine the relationship between exercise and internalizing problems in an adolescent cohort of monozygotic twins and nontwin siblings, regular exercise was both cross-sectionally and longitudinally related to fewer internalizing problems and to an increase in subjective well-being. Interestingly, the finding that exercise related to fewer internalizing problems was supported regardless of the amount of exercise [6], meaning that even low amounts of exercise can be protective against depression and anxiety among adolescents. Furthermore, the odds of experiencing elevated anxiety symptoms regardless of anxiety disorder diagnosis were significantly reduced following physical activity [7]. Given the potential value of exercise as a protective factor against symptoms of depression and anxiety in adulthood [8,9], developing these healthy behaviors early during adolescence is advantageous to both short- and long-term mental health. However, the rise of smartphones and social media use might place some youth at risk for engaging in fewer physical activities and thus greater depression and anxiety throughout their life span.

### Adolescence as the Age of Vulnerability

As adolescence is an important period of development, with unique changes in physical, cognitive, emotional, and social development [10,11], the effect of social media on an individual may be particularly salient during this developmental stage. Whether the benefits of physical activity may mitigate the potentially harmful effects of social media during adolescence remains to be tested. Emotional distress, marked by symptoms of depression and anxiety, has increased congruently with the rise of social media [12], raising the possibility that the two are causally connected. The many effects of social media include harm to the sense of self, behavior that resembles addiction, self-harm promotion, sleep deprivation, fear of missing out, loneliness [13], depression, and anxiety [14-17]. Moreover, the negative effects of social media may have a dose-response relationship, such that adolescents who use a variety of social media platforms and spend the most time on social media are most affected. In one study, adolescents who engaged in multiple social media platforms had sequentially increased likelihoods for depressive and anxiety symptoms based on the number of platforms [15]. Moreover, adolescents who engaged in social media for longer periods had an increased risk of depression and anxiety symptoms [15]. These findings suggest that both the amount of time and breadth of social media platform use relate to symptoms of depression and anxiety in adolescents.

In addition to the developmental changes that occur during adolescence, it is also the period when many internalizing disorders naturally onset [18,19]. Thus, the relationship between social media use and internalizing symptoms may have a bidirectional association such that social media aggravates internalizing psychopathology, and these increases in depression and anxiety symptoms lead adolescents to social media use more often. Negative cognitive styles common in depression and anxiety, such as brooding and rumination, have been found to exacerbate the negative effects associated with social media [20]. A recent study to determine the effect of the frequency of social media use on children and adolescents found that students in grades 7 through 12 with poor mental health use social media with greater frequency than their healthier peers [21]. In a recent systematic review, Keles et al [22] found that time spent, active (posting) and passive (checking, scrolling) use of social media, investment in social media, and social media addiction were all correlated with depression, anxiety, and psychological distress; however, there were considerable methodological constraints across studies, including sampling and measures. Of the 7 studies and meta-analyses that addressed the relationship between symptoms of internalizing disorders (anxiety, depression, and loneliness or fear of missing out) and social media use, 6 studies found positive correlations [13-17,23] and 1 study did not find a relationship [24]. It is also possible that social media has a positive impact on adolescents, including providing educational resources and support to promote mental well-being [25]. Owing to the inconsistency between and limitations of completed studies, it is important to clarify the risk and protective factors that relate to social media use and its influence on internalizing symptoms in adolescents.

### Social Media and Internalizing Symptoms

A better understanding of how social media may be associated with internalizing symptoms differently across adolescence based on gender is also important. The likelihood of adolescent females experiencing depression is 1.5 to 3 times higher than that of adolescent males [18]. Although gender differences in anxiety are not found in childhood, they continue to increase throughout adolescence, with girls experiencing higher levels of anxiety than boys [26]. Furthermore, recent studies have found gender to be a meaningful variable in the relationship between technology use and depressive symptoms. One study showed that the link between technology use and depressive symptoms was stronger for girls than it was for boys, postulating that the negative impacts of self-relevant comparisons might be more threatening to self-worth and therefore more salient to well-being for girls than boys [18]. Importantly, there are also gender differences in physical activity, whereby girls are less active than boys in adolescence [27,28].

### This Study

Although studies have addressed the effect of social media use and physical activity on internalizing disorders separately, to our knowledge, no study to date has explored how both are associated with symptoms of depression and anxiety among adolescents. The aim of this study is to investigate how physical activity interacts with social media use and depression and anxiety symptoms during adolescence. We have two primary hypotheses and several exploratory analyses. First, we hypothesize that social media use would be associated with more internalizing symptoms (ie, depression, anxiety, and loneliness) and less physical activity. Although this hypothesis is based on the literature on physical activity, it is also possible that social media use and physical activity are not related or that more physically active adolescents are also more active on social media. Second, we hypothesize that physical activity would be associated with less severe internalizing symptoms. We also explore gender differences in social media use and internalizing symptoms, especially as they relate to physical activity, and examine differences, if any, between active (posting) and passive (checking) social media use. Finally, we believe that the association between social media and depression would be fully or partially explained by the level of physical activity, such that if adolescents were actively engaged in physical activity, social media use would not be strongly associated with depression. We expect physical activity to have this protective buffering relationship both due to its known associations with depression and anxiety symptoms [5,29] and because it represents an alternative behavior to social media use, indicating a range of extracurricular involvement that is associated with physical and emotional health [30].

## Methods

### Overview

The Institutional Review Boards at the University of Washington and University of Wisconsin approved this study. Data were obtained via a data use agreement between researchers at the University of Wisconsin and Indiana University.

### Participants

Participants were a national sample of adolescents aged 12 to 17 years and their parents or caregivers recruited via Qualtrics panels. Qualtrics recruits panelists with advertisements on the web and conducts a background check to verify their identity before participation. A Qualtrics survey manager recruited adult panel participants between February and March 2019, who indicated that they had adolescent children who spoke English. Parents or caregivers who met these criteria provided information about the survey and an opportunity to provide informed consent for their child’s participation. The minor participants provided consent following parental consent. Adolescents were instructed to complete the survey independently and in a private location. Parents or caregivers completed a panel of surveys assessing social media use, social media rules, and demographic variables, whereas adolescents completed surveys assessing social media, rules around social media, and a variety of psychosocial factors. Adolescents and their caregivers took an average of 39 minutes to complete the survey. Qualtrics parameters were set such that our sample was consistent with race or ethnicity data from the US census [31]. Recruitment procedures were modeled based on prior youth and social media studies using Qualtrics [32,33].

### Materials

#### Assessment of Social Media Use

Adolescents reported how often they checked and how often they posted on social media through a single item question about frequency of checking and a single item about frequency of posting. The social media platform that adolescents reported in this study was Facebook. Scores were on a 9-point frequency range representing “Almost constantly” to “Never” and coded, so that lower scores indicated less social media use and higher scores indicated more social media use. A maximum score of 18 indicated near-constant checking and posting on social media. For our analyses, we not only combined checking and posting into total social media use scores but also conducted some analyses with separate scores for checking and posting to examine differences in types of social media behaviors.

#### Patient Health Questionnaire-9

The Patient Health Questionnaire-9 (PHQ-9) [33] is a widely used screening measure of depression based on *the Diagnostic and Statistical Manual of Mental Disorders* 14 criteria. The nine items are each rated on a 0 (Not at all) to 3 (nearly every day) scale, with higher scores indicating more severe depression symptoms. On the basis of prior research, PHQ-9 scores ≥10 have 88% sensitivity and 88% specificity for major depression. We did not use cutoff scores and viewed depression as a continuous variable, with higher scores representing more severe depression [34]. The internal consistency was excellent (α=.95).

#### Screen for Child Anxiety Related Emotional Disorders

The Screen for Child Anxiety Related Emotional Disorders (SCARED) [34] is a five-item self-report intended to assess anxiety symptoms in children (eg, “I get really frightened for no reason at all”). Scores on each item range from 0 (Not true or hardly ever true) to 2 (True or often true). The five-item SCARED has similar psychometric properties to the original 41-item version, with a sensitivity of 74% and specificity of 73% [35], and we did not use cutoff scores and viewed anxiety as a continuous variable, with higher scores representing more severe anxiety. The internal consistency was good (α=.83).

#### Comprehensive Inventory of Thriving

The Comprehensive Inventory of Thriving (CIT) [36] is a measure of psychological well-being comprised of 18 subscales with 54 items in total, measuring a wide array of components of well-being. We used three items from the Loneliness subscale, comprising prompts such as “I feel lonely” that are rated 1 (Strongly disagree) to 5 (Strongly agree). Higher scores indicate greater loneliness. We also used the CIT Social Support subscale to evaluate adolescents’ feelings of social connectedness and support from others. Of note, this scale does not distinguish between peer social support and parental or other support. This scale is comprised of three prompts such as “There are people I can depend on to help me” that are rated 1 (Strongly disagree) to 5 (Strongly agree). Higher scores indicated greater social support in our sample. The CIT has excellent psychometric properties and good convergent validity with existing measures of psychological well-being [37]. Internal consistency was excellent for loneliness (α=.90) and good for social support (α=.88).

#### Assessment of Physical Activity and Other Extracurricular Activities

Physical activity was assessed with three questions rated on a 1 (Never) to 5 (4 or more times a week) scale, with higher scores indicating more physical activity. Questions assessed how often, outside of school hours, the adolescents exercised to the point of being out of breath or sweating with or without club associations, and as a part of competitive sports. Internal consistency was good (α=.81).

In addition to assessing physical activities, involvement in other extracurricular activities was assessed with four items rated on a 1 (0 hours) to 5 (11 or more hours) point scale. Higher scores indicated more time spent on extracurricular activities. Questions assessed time involvement in music (band, choir, orchestra, lessons, and practicing), clubs outside of school, clubs at school, and time playing sports on a school team. Importantly, the time spent on a school sports team was positively associated with the assessment of total physical activity, as described earlier (*r*=0.60; *P*<.001). Thus, as we wanted to examine the relationship between social media use and internalizing psychopathology with physical activity as a potential mediator, we removed the sports items from the other extracurricular items and instead included items assessing the time spent on music, school clubs, and out-of-school clubs. With both the sports item included and the sports item removed, internal consistency was acceptable and did not change (α=.74).

### Data Analyses

Analyses were conducted using R Studio [38]. We used the *psych* package to examine the direct relationships between social media use (checking, posting, and total) and internalizing psychopathology (depression, anxiety, and loneliness). Mediation analyses for variables of interest (physical activity and loneliness) were conducted in the *mediation* package [39] using *diagram* to plot results. Of note, this package uses the more recent bootstrapping method of Preacher and Hayes [40] to address the power limitations of the Sobel test. Standardized regression coefficients were determined using the *lm.beta* package. For all analyses, we corrected for multiple comparisons using the Bonferroni method. Effect sizes were interpreted according to Cohen standards [41].

## Results

### Descriptive Statistics and Correlations

A total of 4592 adolescent-parent dyads completed the survey. The average age of adolescent participants was 14.62 (SD 1.68; range 12-17) years. Most adolescent participants were White (3070/4592, 66.86%), non-Hispanic (3703/4592, 80.73%) with White (3148/4592, 68.55%), and non-Hispanic parents or caregivers (3798/4592, 82.71%). More adolescent males (2392/4592, 52.09%) than adolescent females completed the survey, whereas most parents or caregivers completing the survey were female (2652/4592, 57.75%). Most parents or caregivers identified being biological parents of the adolescent (3934/4592, 85.67%), followed by stepparents (246/4592, 5.36%). On the basis of parents’ self-reported household socioeconomic status, the majority of the sample was above the poverty line (3402/4592, 74.09%). Table 1 presents detailed demographic information.

**Table 1:** Participant characteristics.

Variable			Parent report, n (%)		Adolescent report, n (%)
**Relationship to child in research (n=4581)**					
	Biological parent	3934 (85.88)		—^a^	
	Stepparent	246 (5.37)		—	
	Parent’s partner (living together)	137 (2.99)		—	
	Adoptive parent	120 (2.61)		—	
	Foster parent	12 (0.26)		—	
	Grandparent	106 (2.31)		—	
	Other relative or guardian	26 (0.57)		—	
**Number of children (n=4588)**					
	1	1357 (29.58)		—	
	2	1745 (38.03)		—	
	3	872 (19)		—	
	4	334 (7.27)		—	
	5	147 (3.2)		—	
	≥6	133 (2.9)		—	
**Child participating in research (n=4565)**					
	Youngest child	1456 (31.89)		—	
	Middle child	632 (13.84)		—	
	Oldest child	1584 (34.7)		—	
	Only child	893 (19.56)		—	
**Gender (n=4592)**					
	Female	2652 (57.75)		2130 (46.39)	
	Male	1877 (40.88)		2392 (52.09)	
	Nonbinary	26 (0.57)		23 (0.5)	
	Female to male transgender	20 (0.43)		25 (0.54)	
	Male to female transgender	5 (0.11)		5 (0.11)	
	Other	3 (0.07)		0 (0)	
	Prefer not to answer	9 (0.2)		17 (0.2)	
**Ethnicity (n=4592)**					
	Not Hispanic, Latino, or Spanish origin	3798 (82.71)		3707 (80.73)	
	Mexican American, Chicano	386 (8.41)		439 (9.56)	
	Puerto Rican	166 (3.61)		178 (3.88)	
	Cuban	44 (0.96)		50 (1.09)	
	Another Hispanic, Latino, or Spanish origin	148 (3.22)		152 (3.31)	
	Prefer not to answer	50 (1.09)		66 (1.44)	
**Race (n=4592)**					
	White or Caucasian	3148 (68.55)		3070 (66.86)	
	Black or African American	674 (14.68)		699 (15.22)	
	American Indian or Alaska Native	123 (2.68)		116 (2.52)	
	Asian	230 (5.01)		211 (4.59)	
	Asian Indian	17 (0.37)		17 (0.37)	
	Other Asian	7 (0.15)		7 (0.15)	
	Native Hawaiian or other Pacific Islander	42 (0.91)		36 (0.78)	
	Multiracial	146 (3.18)		221 (4.81)	
	Other	36 (0.78)		31 (0.68)	
	Prefer not to answer	72 (1.57)		82 (1.79)	
	Latino, Hispanic, or Mexican	97 (2.11)		102 (2.22)	
**Highest completed education (n=4592)**					
	4th grade	—		1 (0.02)	
	5th grade	—		19 (0.41)	
	6th grade	—		489 (10.65)	
	7th grade	—		644 (14.02)	
	8th grade	—		747 (16.27)	
	9th grade	—		776 (16.9)	
	10th grade	—		828 (18.03)	
	11th grade	—		681 (14.83)	
	12th grade	—		361 (7.86)	
	Other	—		12 (0.26)	
	Prefer not to answer	—		23 (0.5)	
	College	—		11 (0.24)	
**Highest completed education (n=4592)**					
	High school incomplete or less	292 (6.36)		—	
	High school graduate or General Educational Development	886 (19.29)		—	
	Some college	1440 (31.36)		—	
	Four-year college degree or bachelor’s degree	1049 (22.84)		—	
	Some postgraduate or professional schooling	176 (3.83)		—	
	Postgraduate or professional degree	718 (15.64)		—	
	Prefer not to answer	31 (0.66)		—	

^a^For some questionnaires, only one party (adolescent or parent) filled it out.

With regard to total social media use, 7.14% (328/4592) of our sample indicated never checking or posting on social media, whereas 11.26% (517/4592) reported being on social media “almost constantly.” The mean checking score was closest to “A few times/day,” whereas the mean posting score was approximately “Once a day.” There was a significant positive relationship between checking and posting (*r*=0.72; *P*<.001). The average SCARED score was 2.32 (SD 2.54; range 0-10), which approaches clinical risk for an anxiety disorder. The average PHQ-9 score in our sample was 5.43 (SD 6.90; range 0-27), indicating mild depression.

In our sample, the average physical activity to the point of being out of breath or sweating was about twice a week with noncompetitive physical activities and competitive sports, both averaging about twice a week. Thus, the overall mean for physical activity, calculated by totaling all three items and dividing by three, was approximately 2.93 (SD 3.74), indicating that total physical activity was about twice a week. Of note, this is lower than the national recommendations for physical activity in adolescents [16]. With regard to involvement in extracurricular activities, with the sports item removed, the summed score was 5.94, indicating an average of 3-5 hours per week of extracurricular activities. When we included the sports item with the other three, the summed score was 8.22, also indicating 3-5 hours per week of extracurricular activities on average.

Descriptive statistics of the primary variables of interest, including demographic information, are presented in Table 1. Table 2 presents a correlation matrix.

**Table 2:** Correlation matrix.

Variable			Age		Depression		Anxiety		Loneliness		Social support		Physical activity		Extracurricular		Social media checking		Social media posting
**Age**																			
	*r*	—^a^		—		—		—		—		—		—		—		—	
	*P* value	—		—		—		—		—		—		—		—		—	
**Depression**																			
	*r*	−0.08		—		—		—		—		—		—		—		—	
	*P* value	<.001		—		—		—		—		—		—		—		—	
**Anxiety**																			
	*r*	−0.12		0.73		—		—		—		—		—		—		—	
	*P* value	<.001		<.001		—		—		—		—		—		—		—	
**Loneliness**																			
	*r*	−0.04		0.67		0.61		—		—		—		—		—		—	
	*P* value	.006		<.001		<.001		—		—		—		—		—		—	
**Social support**																			
	*r*	0.05		−0.32		−0.22		−0.31		—		—		—		—		—	
	*P* value	<.001		<.001		<.001		<.001		—		—		—		—		—	
**Physical activity**																			
	*r*	−0.07		0.10		0.09		−0.03		0.06		—		—		—		—	
	*P* value	<.001		<.001		<.001		.02		<.001		—		—		—		—	
**Extracurricular**																			
	*r*	−0.08		0.43		0.42		0.28		−0.09		0.41		—		—		—	
	*P* value	<.001		<.001		<.001		<.001		<.001		<.001		—		—		—	
**Social media checking**																			
	*r*	0.12		0.25		0.20		0.19		−0.02		0.12		0.17		—		—	
	*P* value	<.001		<.001		<.001		<.001		—		<.001		<.001		—		—	
**Social media posting**																			
	*r*	0.06		0.34		0.28		0.24		−0.11		0.17		0.28		0.72		—	
	*P* value	<.001		<.001		<.001		<.001		<.001		<.001		<.001		<.001		—	
**Social media total**																			
	*r*	0.10		0.32		0.26		0.23		−0.08		0.15		0.25		0.92		0.93	
	*P* value	<.001		<.001		<.001		<.001		<.001		<.001		<.001		<.001		<.001	

^a^Not applicable.

As expected, there was a significant positive relationship between depression and anxiety in adolescents, such that higher depression scores were associated with higher anxiety scores (*r*=0.73; *P*<.001). The relationships between internalizing symptoms and social media behavior were significant, such that higher depression (*r*=0.32; *P*<.001) and anxiety (*r*=0.26; *P*<.001) scores were associated with more social media use (Table 2).

Social support was inversely related to depression (*r*=−0.32; *P*<.001), anxiety (*r*=−0.22; *P*<.001), loneliness (*r*=−0.31; *P*<.001), and total time spent on social media (*r*=−0.08; *P*<.001). When tested on a linear model, social support was a significant predictor of lower depression (multiple *R^2^*=0.18; *F*_2,4466_=517.20; *P*<.001; *f*^2^=0.22) and anxiety (multiple *R^2^*=0.11; *F*_2,4459_=262.30; *P*<.001; *f*^2^=0.11). These effects were medium and small, respectively; were statistically significant; and survived the Bonferroni correction for multiple comparisons. However, there was no significant interaction between social media checking and social support for depression (*P*=.30) or anxiety (*P*=.83). This was also true for the interaction between social media posting and social support for depression (*P*=.46) and anxiety (*P*=.25)

Contrary to expectations, there was a significant positive relationship between depression and physical activity scores (*r*=0.10; *P*<.001) and depression and other extracurricular activities (*r*=0.40; *P*<.001), such that adolescents with higher depression scores reported higher levels of physical activity and extracurricular activities. We proceeded to investigate group differences that may account for this and test mediations as planned, despite the unexpected direction of the relationship between physical activity and internalizing symptoms.

### Investigating Group Differences

#### Age-Related Differences

We conducted a series of regressions to examine the relationships among internalizing symptoms, social media use, and physical activity, controlling for other variables including age and gender. First, we examined the nature of the relationships between depression or anxiety scores and age and social media and age. As was previously shown in correlations (Table 2), there was a significant relationship between age and depression (multiple *R^2^*=0.01; *F*_1,4500_=25.56; *P*<.001; *f*^2^=0.01), age and anxiety (multiple *R^2^*=0.01; *F*_1,4500_=25.56; *P*<.001; *f*^2^=0.01), and age and social media use (multiple *R^2^*=0.01; *F*_1,4555_=44.41; *P*<.001; *f*^2^=0.01), and older adolescents showed more social media use than younger adolescents, and depression and anxiety symptoms decreased with age. Of note, although regression results were statistically significant and survived Bonferroni correction, the effect sizes were small.

#### Gender Differences

We wanted to characterize gender differences in our sample before controlling for gender as a covariate in our regression analyses. Owing to the nonnormal distribution of the PHQ-9 and SCARED data, we could not compare all genders using analysis of variance. Thus, to further investigate these symptom differences between genders, we first used Kruskal-Wallis tests and found significant differences among all gender categories represented (female, male, nonbinary, female to male transgender, and male to female transgender) for depression (Kruskal-Wallis χ^2^_5_=42.4; *P*<.001), anxiety (Kruskal-Wallis χ^2^_5_=55.0; *P*<.001), loneliness (Kruskal-Wallis χ^2^_5_=37.7; *P*<.001), and total social media use (Kruskal-Wallis χ^2^_5_=27.2; *P*<.001). We tested this further by subsetting our data and comparing males to females, and then by grouping males and females into one group and all other gender categories into a second group to conduct a series of Welch two-tailed *t* tests. There were significant differences in depression scores based on the majority gender, with males reporting significantly higher depression scores (*t*_4444_=−4.51; *P*<.001) and more loneliness (*t*_4480_= −2.31; *P*=.02) than females, but there were no differences in anxiety scores (*t*_4443_=0.76; *P*=.44) between men and women. Women reported significantly more social media use than males (*t*_4499_=4.47; *P*<.001). Males reported more physical activity than females (*t*_4397_=−9.89; *P*<.001). Both males and females (grouped together) showed significantly lower (less severe) internalizing scores than nonbinary gender or transgender adolescents (depression: *t*50=−6.48, *P*<.001; anxiety: *t*_52_=−6.68, *P*<.001; loneliness: *t*_53_=−6.80, *P*<.001), with no differences in total physical activity (*t*_53_=1.26; *P*=.21). Interestingly, although there were no significant differences in total social media use between males and females and nonbinary and transgender groups (*t*_55_=−0.93; *P*=.36), male and female adolescents reported significantly more posting (*t*_55_=3.80; *P*<.001), whereas nonbinary and transgender adolescents reported significantly more checking on social media (*t*_53_=−2.29; *P*=.03). Finally, we assessed whether the effects of social media use on internalizing symptoms were moderated by gender (male vs female) and found significant results for all internalizing symptoms, including depression (*b*=0.28, SE 0.04; *P*<.001), anxiety (*b*=0.11, SE 0.02; *P*<.001), and loneliness (*b*=0.10, SE 0.02; *P*<.001). The associations between social media use and internalizing symptom severity were stronger in males than in females.

#### Controlling for Age and Gender Covariates

Given the significance of age and gender in our models, we wanted to control for these variables in subsequent analyses. Using gender and age as covariates, we tested the associations between different types of social media use (total, checking, and posting) and internalizing symptoms in linear regressions. The reported *R^2^* values represent multiple *R^2^* values for the tested linear model. Total social media use was associated with more depressive symptoms (*R^2^*=0.12; *F*_3,4480_=207.1; *P*<.001; *f*^2^=0.14), anxiety (*R^2^*=0.09; *F*_3,4477_=145.6; *P*<.001; *f*^2^=0.10), and increased loneliness (*R^2^*=0.06; *F*_3,4512_=98.06; *P*<.001; *f*^2^=0.06), controlling for age and gender. Contrary to predictions, total social media use was associated with more physical activity (*R^2^*=0.04; *F*_3,4529_=67.78; *P*<.001; *f*^2^=0.04). In examining differences between types of social media behavior, both checking and posting were significantly associated with more depression (checking: *R^2^*=0.08; *F*_3,4492_=138.1; *P*<.001; *f*^2^=0.09; posting: *R^2^*=0.13; *F*_3,4485_=222.6; *P*<.001; *f*^2^=0.15), more anxiety (checking: *R^2^*=0.06, *F*_3,4489_=97.74, *P*<.001, *f*^2^=0.06; posting: *R^2^*=0.10, *F*_3,4482_=159.6, *P*<.001, *f*^2^=0.11), and more loneliness (checking: *R^2^*=0.04, *F*_3,4524_=69.23, *P*<.001, *f*^2^=0.04; posting: *R^2^*=0.06, *F*_3,4518_=102.0, *P*<.001, *f*^2^=0.06), controlling for age and gender and correcting for multiple comparisons using Bonferroni methods. The effect sizes ranged from small to medium. As evidenced by these results and examining β coefficients and effect sizes between regression models, there were no major differences when comparing social media posting versus social media checking. As such, we proceeded by using social media total as our independent variable in the remaining analyses.

Owing to the unexpected findings regarding the relationship between physical activity and depression and anxiety symptoms based on correlations, we wanted to further examine differences related to age and gender that may account for these relationships. We regressed internalizing symptoms (depression, anxiety, and loneliness) on total physical activity, controlling for other extracurricular activities, age, and gender. This was significant in the hypothesized direction: more physical activity predicted decreased depression, anxiety, and loneliness symptoms when controlling for other nonsports-related extracurricular activity, age, and gender (depression: *R^2^*=0.20, *F*_4,4440_=273.1, *P*<.001, *f*^2^=0.25; anxiety: *R^2^*=0.19, *F*_4,4437_=257.6, *P*<.001, *f*^2^=0.23; loneliness: *R^2^*=0.11, *F*_4,4472_=134.8, *P*<.001, *f*^2^=0.22). All the effect sizes were medium. When we controlled for social media use as an additional covariate, the relationship between physical activity and internalizing symptoms became even stronger (depression: *R^2^*=0.25, *F*_5,4421_=299.0, *P*<.001, *f*^2^=0.33; anxiety: *R^2^*=0.22, *F*_4,4418_=251.7, *P*<.001, *f*^2^=0.28; loneliness: *R^2^*=0.14, *F*_5,4452_=147.6, *P*<.001, *f*^2^=0.16). Thus, although the relationship between social media and internalizing remains significant in the model, with more social media use linked to more depression and anxiety symptoms, physical activity showed a significant association in the opposite direction, as our hypotheses and prior research in adults would suggest. The effect sizes were medium and survived the Bonferroni corrections.

### Mediation Analyses

**Figure 1:**
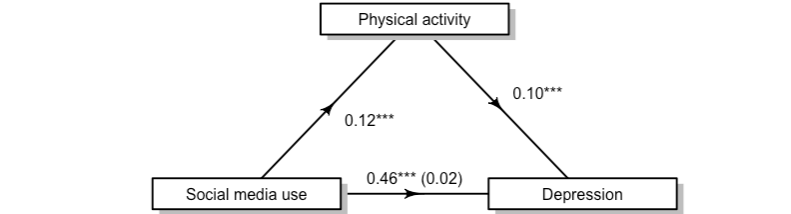
The relationship between social media use and depression is partially mediated by physical activity. ***Statistical significance at *P*<.001.

The effect of total social media use on depression is partially mediated by physical activity. As Figure 1 illustrates, the regression coefficient between total social media use and depression severity and the regression coefficient between total physical activity and depression severity were significant. The indirect effect is (0.12) × (0.10) = 0.01. We tested the significance of this indirect effect using bootstrapping. Unstandardized indirect effects were computed for each of the 500 bootstrapped samples, and the 95% CI was computed by determining the indirect effects at the 2.5th and 97.5th percentiles. Thus, the bootstrapped unstandardized indirect effect was 0.01 (95% CI 0.01-0.02). Although small, the indirect effect was statistically significant (*P*<.001)*.* Here, physical activity accounted for 2.6% (95% CI 1%-4%) of the effect of total social media use on depressive symptoms. Of note, after examining total social media use, we ran mediations with checking and posting behaviors. We observed the same pattern of findings in these mediations; therefore, we report only on total social media use.

**Figure 2:**
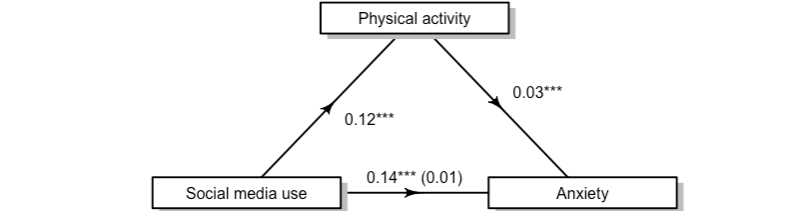
The relationship between social media use and anxiety is partially mediated by physical activity. ***Statistical significance at *P*<.001.

We observed the same pattern of findings in examining the relationship between total social media use, anxiety, and physical activity (Figure 2). The results showed that more social media use was associated with higher anxiety symptoms and that more physical activity was associated with greater anxiety. Physical activity partially mediated the relationship between social media use and anxiety, again using 500 bootstrapped samples. The indirect effect was very small (0.12) × (0.03) = 0.004 (95% CI 0.002-0.01), but statistically significant (*P*<.001)*.* Here, physical activity accounted for 3% (95% CI 1%-5%) of the effect of total social media use on anxiety symptoms.

**Figure 3:**
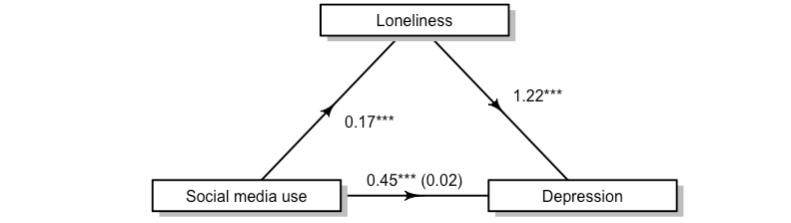
The relationship between social media use and depression is partially mediated by loneliness. ***Statistical significance at *P*<.001.

We continued to examine the complex relationship between social media and internalizing by exploring loneliness as a mediator. We hypothesized that feelings of loneliness would account for social media’s relationship with increased depression, and indeed, this was true (Figure 3). Using 500 bootstrapped samples, the unstandardized indirect effect of loneliness was (1.22) × (0.17) = 0.21 (95% CI 0.18-0.23; *P*<.001). Here, loneliness accounted for a large proportion of the effect of total social media use on depressive symptoms (45%; 95% CI 41%-50%).

## Discussion

### Principal Findings

Consistent with the current literature [17,42], we found that more social media use was associated with higher levels of internalizing symptoms (depression, anxiety, and loneliness). We also found that more social media use was associated with greater physical activity. Given our cross-sectional design, it is unclear whether social media use leads to more depression, anxiety, and loneliness or if these internalizing symptoms cause individuals to seek out more social media, which could feed into a vicious cycle. As anticipated, depression and anxiety were positively associated with one another in adolescents [5]. There is substantial evidence that physical activity is associated with lowered rates and decreased severity of internalizing symptoms in adults [7-9] and promising evidence that such a relationship exists for adolescents [5]. Our results showed that physical activity was associated with less severe symptoms of depression and anxiety. Our findings regarding physical activity were further explored in the mediation analyses. The results of our mediation analyses suggest a small but significant mediation of physical activity on the relationship between social media use and anxiety and depression. The mediating effects were greater for depression, which could partially be due to the correlation between depression and other variables, including loneliness and gender.

Initially, when examining bivariate correlations in our data, the findings seem to conflict with the existing literature [43-45] and the common sense idea that physical activity is associated with better health outcomes, including reduced anxiety and depression [46]. However, when our model controlled for other variables including age, gender, and extracurricular activities, which all independently had significant associations with depression, our findings were in line with our hypotheses. The various reasons adolescents in our sample engaged in physical activity remain unknown. Adolescents with depression and anxiety may have been trying to exercise more to improve their mood, and we expected that social media (likely a sedentary behavior) would replace physical activity. However, in some adolescents and teenagers, physical activity may not be driven by health benefits but instead fueled by social media itself: comparison to others and using social media as fitness inspiration (*fitspo*). Indeed, fitspo in adults has been linked to negative mental health outcomes, including poor appearance-based self-perception in some studies [47,48] but not all [49,50], but less is known about this trend in adolescents and young adults. Adolescence is also a time of many physical changes (ie, higher fat-to-muscle ratio among girls), along with the development of social-cognitive processes from others’ perspectives. Specifically, adolescents become keenly aware of others’ perspectives and their bodies [51]. Moreover, in predicting that physical activity would act as a buffer between potentially harmful social media use and internalizing symptoms, we drew primarily from the adult literature. In adults, physical activity has been used as an intervention for depression and anxiety, but less is known about the use of exercise interventions in depressed and anxious youth. However, a recent meta-analysis suggested that exercise interventions may be associated with decreases in adolescent depression [52]. Although this study was not an exercise intervention study and cannot draw conclusions regarding interventions, it is recommended that future studies investigate exercise interventions in adolescents, particularly given the number of adolescents who are prescribed psychotropic medications [53,54] despite their harmful effects.

Additional implications can be drawn from the mediation results and regression analyses. With regard to physical activity partially mediating the effect of social media on depression and anxiety, we can surmise that it is not simply checking or posting on social media that is linked with depression because there is an indirect effect of physical activity such that more physical activity also accounts for the connection between social media and internalizing symptoms in our sample. This may indicate that the amount of overall activity, physical activity, social media engagement, and extracurricular activity may be added to allostatic load. It is also important to consider the idea that physical activity in the form of competitive sports may increase depression and anxiety, as prior studies have shown that athletes are vulnerable to a range of mental health problems [55]. Although physical activity and extracurricular activities are generally believed to be a positive activity for adolescents at risk for depression and anxiety, this may not be true for all adolescents [56]. Supplementing or replacing physical activity and extracurricular activities with other well-being activities, including socializing in person and making time for leisure, relaxation, or other value-driven behaviors is important to consider. One implication of our findings is that if social media use continues to increase [57,58], doing more physical activity or extracurricular activities cannot entirely mitigate its potentially harmful effect. However, given our finding of the unique link between physical activity and internalizing symptoms when controlling for social media and other activities, physical activity should continue to be researched as a potential intervention in depressed and anxious adolescents, perhaps combined with setting limits on social media and reducing other extracurricular activities.

Our results also have implications for future studies. The gender differences in this study indicate that this is an important area to account for: male adolescents in this study presented higher scores in depression and loneliness, whereas female adolescents checked social media more. Considering the positive relationship between social media use and internalizing symptoms, it is clear that no one size fits all approaches. More research is needed on transgender and nonbinary adolescents. Furthermore, given the relationships between social media, depression, and loneliness, it may be important to target social connectedness beyond social media. A potential method for doing this could encourage adolescents to combine socializing and physical activity. It is also possible and likely that the type of social media platform matters, so continuing to investigate these phenomena across different types of media accounts is important.

This study has several strengths. First, our study used a large, nationally representative sample that allowed the study to be adequately powered to conduct analyses and draw generalizable conclusions. Second, with survey data using brief and reliable measures, we were able to capture a variety of variables in a short period, from social media use (checking and posting) to internalizing symptoms, social support, extracurricular activities, and physical activity. To the best of our knowledge, this is the largest data set capturing adolescent mental health, social media, and physical activity in a nationally representative sample of adolescents.

### Limitations

Despite the strengths of this study, including the large sample size and diversity of respondents, there were several limitations. First, there is often concern regarding the validity of adolescent self-report, as adolescents’ self-perception and presentation can be influenced by many factors, including social desirability and the sensitivity of items being reported [59]. We attempted to control for this by instructing adolescents to complete the survey independently and in a private location from their parents. Second, physical activity is often overreported due to the perceived social desirability of the activity [60]. Studies have found fallibility in the perception of time, frequency, and intensity of activities such as driving and web-based activities. Similarly, students have been found to overreport social media use by an average of two hours. There is also a significant error in the self-report of social media use in adults. As people spent more time on Facebook, reports of time spent were less accurate [61]. Third, this study is cross-sectional; therefore, causality and directionality of effects cannot be stated. Fourth, it is possible that adolescents use social media while exercising, which could complicate the interpretation of findings. Finally, our effect sizes ranged from small to medium, and replication was recommended.

### Conclusions

In conclusion, this study showed that when controlling for social media use, physical activity is associated with decreased internalizing symptoms in a nationally representative sample of adolescents. We did not observe a significant association between physical activity and reduced depression and anxiety until after controlling for social media use and other extracurricular activities, which may imply that if adolescents are involved in multiple activities, the protective mental health benefits of physical activity can decrease. As this was a cross-sectional study and a direct causal relationship between time on social media and internalizing symptoms cannot be established, the type of social media use should be more specifically examined in future work [61]. Perhaps an intervention that promotes physical activity in social groups in person, or social media, is the next step for adolescents with depression and anxiety symptoms. Physical activity that facilitates feelings of connectedness would likely help teenagers at risk for depression, anxiety, and loneliness; however, this is an empirical question that should be tested. Additional future work should use multiple report sampling to learn more about parental physical activity and the modeling of health behaviors. Finally, objective tracking of social media use (through partnerships with industry) and objective physical activity monitoring in adolescents via passive sensors such as a Fitbit is recommended to fully understand these relationships.

## Abbreviations

CIT: Comprehensive Inventory of Thriving
PHQ-9: Patient Health Questionnaire-9
SCARED: Screen for Child Anxiety Related Emotional Disorders
